# Endometriosis and Organochlorinated Environmental Pollutants: A Case–Control Study on Italian Women of Reproductive Age

**DOI:** 10.1289/ehp.0800273

**Published:** 2009-03-31

**Authors:** Maria Grazia Porpora, Emanuela Medda, Annalisa Abballe, Simone Bolli, Isabella De Angelis, Alessandro di Domenico, Annamaria Ferro, Anna Maria Ingelido, Antonella Maggi, Pierluigi Benedetti Panici, Elena De Felip

**Affiliations:** 1 Department of Gynaecology and Obstetrics, Sapienza University of Rome, Rome, Italy; 2 National Centre for Epidemiology, Surveillance and Health Promotion, National Institute for Health, Rome, Italy; 3 Department of the Environment and Primary Prevention, National Institute for Health, Rome, Italy; 4 Service for Biotechnology and Animal Welfare, National Institute for Health, Rome, Italy

**Keywords:** biomonitoring, case-control study, dioxins, endometriosis, PCBs

## Abstract

**Background:**

Endometriosis is a common gynecologic disease characterized by the ectopic growth of endometrial tissue. In industrialized countries, it affects approximately 10% of women of reproductive age. Its etiology is unclear, but a multifactorial origin is considered to be most plausible. Environmental organochlorinated persistent pollutants, in particular dioxins and polychlorinated biphenyls (PCBs), have been hypothesized to play a role in the disease etiopathogenesis. However, results of studies carried out on humans are conflicting.

**Objective:**

We evaluated the exposure to organochlorinated persistent pollutants as a risk factor for endometriosis.

**Methods:**

We conducted a case–control study in Rome on 158 women comprising 80 cases and 78 controls. In all women, serum concentrations of selected non-dioxin-like PCBs (NDL-PCBs) and dioxin-like PCBs (DL-PCBs), 1,1-dichloro-2,2,-*bis*(4-chlorophenyl)-ethene (*p,p*′-DDE), and hexachlorobenzene (HCB) were determined by ion-trap mass spectrometry. DR-CALUX bioassay was employed to assess the 2,3,7,8-tetrachlorodibenzo-*p*-dioxin toxicity equivalent (TEQ) concentrations of polychlorinated dibenzo-*p*-dioxins (PCDDs), polychlorinated dibenzofurans (PCDFs), and DL-PCBs.

**Results:**

We found an increased risk of endometriosis for DL-PCB-118 [odds ratio (OR) = 3.79; 95% confidence interval (CI), 1.61–8.91], NDL-PCB-138 (OR = 3.78; 95% CI, 1.60–8.94), NDL-PCB-153 (OR = 4.88; 95% CI, 2.01–11.0), NDL-PCB-170 (OR = 3.52; 95% CI, 1.41–8.79), and the sum of DL-PCBs and NDL-PCBs (OR = 5.63; 95% CI, 2.25–14.10). No significant associations were observed with respect to HCB or to the sum of PCDDs, PCDFs, and DL-PCBs given as total TEQs.

**Conclusions:**

The results of this study show that an association exists between increased PCB and *p,p*′-DDE serum concentrations and the risk of endometriosis.

The possible role of the exposure to environmental chemicals as a co-causal factor in the etiology of endometriosis has been the object of scientific debate in the last 20 years. Endometriosis is a common gynecologic disease characterized by the ectopic growth of endometrial tissue and is often associated with pelvic pain and/or infertility. It affects approximately 10% of women of reproductive age in Italy as well as in other industrialized countries (Eskenazi and Warner 1997; Gruppo Italiano per lo Studio dell’Endometriosi 1994), and its prevalence and severity are reported to be increasing in developing countries (Donnez et al. 2002).

Its etiology is unclear, although a multifactorial origin, resulting from the contribution of immunologic, genetic, and environmental factors, is considered to be most plausible. The hypothesis that exposure to immunotoxic endocrine-disrupting environmental pollutants could play a role in the disease etiology first arose from the study of Rier and coworkers (Rier et al. 1993). These authors observed a dose-dependent increase of incidence and severity of spontaneous endometriosis in a colony of monkeys chronically exposed to dioxin [2,3,7,8-tetrachlorodibenzo-*p*-dioxin (2,3,7,8-TCDD)], the most toxic member of the family of polychlorinated dibenzo-*p*-dioxins (PCDDs) and polychlorinated dibenzofurans (PCDFs), 210 different molecules or congeners generally referred to as dioxins. Although criticized by some scientists (Guo 2004; Hitchin 1994), this study opened the way to a number of studies on the relationship between the disease and environmental pollutants. Major criticism involved the incidental nature of the observation—endometriosis was not a prospectively defined end point of the experiment, and its presence in monkeys was observed many years after the end of the treatment—and appropriateness of statistical analysis of the results. The small sample size (24 animals assigned to three groups of 8 animals each), the limited values of dioxin exposure levels (a low-dose and a high-dose group), and the use of linear regression and *t*-test based on normality assumption were the study elements deemed to be more critical in interpreting study results. Together with dioxins, polychlorinated biphenyls (PCBs), a family of persistent and bioaccumulative industrial compounds widely used until the 1980s, have been the pollutants principally investigated as to their possible role in the disease onset or progression. In the general population, diet accounts for over 90% of total exposure to dioxins and PCBs.

PCBs comprise 209 different congeners which, according to the structure, are grouped into 12 dioxin-like PCB (DL-PCB) congeners, with no or only one chlorine in the *ortho* position, and non-dioxin-like PCBs (NDL-PCBs), characterized by the presence of two or more chlorines in the *ortho* positions. The latter are normally much more abundant than DL-PCBs in environmental, food, and human specimens. The most abundant congeners in human tissues include NDL-PCBs 28, 52, 101, 138, 153, and 180—referred to as ‘‘indicators’’ (Appel 2003) because conventionally they are used to estimate the overall PCB content in specimens of biological origin (EFSA 2005)—along with a few others such as DL-PCB-105, DL-PCB-118, DL-PCB-156, and DL-PCB-167, and NDL-PCB-170, PCB-138, PCB-153, and PCB-180, are prevalent in all human tissues and account for 50–80% of total PCB content in serum (Glynn et al. 2000).

DL-PCBs have been the first PCB congeners to be considered in association with endometriosis because, as dioxins, they bind to the aryl hydrocarbon receptor (AhR) and elicit the same spectrum of toxic activities through the same mechanism of action. Indeed, DL-PCBs, rather than dioxins, were suggested to be associated with an endometriotic effect (Rier et al. 2001) when blood samples from exposed monkeys were analyzed years after the experiment was completed and significant concentrations of these compounds were detected, possibly originating from contaminated food.

On the epidemiologic side, several studies have been conducted to investigate the potential relationship between endometriosis and dioxins and/or PCBs, including the non-dioxin-like congeners, but their results are conflicting. Major differences in study design, analytical methods, and the number and kinds of congeners measured make comparability (if any) between different studies limited, as recently discussed by some authors (Anger and Foster 2008; Heilier et al. 2008). In the last decade, the hypothesis of a correlation between the disease and environmentally persistent organohalogenated compounds has been extended to include other organohalogenated pollutants present in human tissues such as polybrominated biphenyls (PBBs) (Hoffman et al. 2007), hexachlorobenzene (HCB), and 1,1-dichloro-2,2-*bis*(4-chlorophenyl)-ethene (*p,p*′-DDE) (Lebel et al. 1998; Tsukino et al. 2005), all characterized by endocrine-disrupting and immunotoxic activity (Agency for Toxic Substances and Disease Registry 2002; Blanck et al. 2000; Davis et al. 2005; Foster et al. 1992, 1995; Halloway et al. 2005; Reed et al. 2007; Windham et al. 2005). No significant association for these pollutants has been evidenced in the studies performed to date.

In previous studies carried out to investigate a possible association between organochlorinated compounds and endometriosis, we found significantly higher concentrations of some of the most abundant PCBs and higher concentrations of *p,p*′-DDE, in women affected by the disease (Porpora et al. 2006; Quaranta et al. 2006). We also observed that such increased concentrations were associated with altered natural killer (NK) immune responses (Quaranta et al. 2006). On the contrary, no increase in blood concentrations of dioxin-like chemicals (PCDDs, PCDFs, and the sum of the 12 DL-PCBs) was observed in women affected by the disease at different degrees (De Felip et al. 2004).

The present study was funded by Italy’s Ministry of Health and National Institute for Health in the context of research activities aimed to characterize the risk for women’s reproductive health from exposure to persistent organic pollutants of environmental origin. It is the largest study carried out in Italy on the possible association between endometriosis and persistent organochlorinated pollutants of high toxicologic relevance.

## Methods

### Patients

Of the 312 women who underwent laparoscopy between January 2002 and December 2005 at the Department of Gynaecology and Obstetrics, Policlinico Umberto I, University of Rome Sapienza, for endometriosis or other benign gynecologic conditions, 158 patients were enrolled in the study. The vast majority of women participating in the study underwent surgery between March 2004 and October 2005. The protocol of this study was approved by the Sapienza University, School of Medicine, Institutional Review Board. All patients met the inclusion criteria: 18–45 years of age, residence in Rome in the last 5 years, no breast-feeding history, absence of immunologic, hormonal disorders, or chronic diseases, and no occupational exposure to PCBs or pesticides.

All enrolled women signed an informed consent form. A physician unaware of the indications to laparoscopy administered a questionnaire before surgery which documented age, education, job, medical, gynecologic and obstetric history, height and weight, and smoking and dietary habits. The questionnaire was designed to obtain information on potential confounders, including metabolic diseases, gravidity, parity, and weight changes in the last years. A detailed medical and gynecologic history was taken, and all patients underwent clinical and ultrasound examinations. For each woman, the body mass index (BMI) was calculated. Before laparoscopy, a blood specimen of approximately 30 mL was collected from the cubital vein in Vacutainer tubes and centrifuged. Serum specimens were stored at −20°C until subjected to analysis. A 10-mm laparoscopy was performed under general anesthesia. The presence of endometriosis was confirmed by histologic analysis of lesions, and the disease was staged in 80 women according to the revised American Society of Reproductive Medicine (ASRM) classification. The control group consisted of women without complaints of infertility or pelvic pain who were undergoing laparoscopy for benign gynecologic conditions and had no visual evidence and histologic features of endometriosis in random peritoneal biopsies.

The questionnaire administered to document dietary habits included 16 questions on the frequency of consumption (times/month) of various milk, meat, and fish products.

### Analysis of serum samples: polychlorobiphenyls and pesticides

Serum samples were added with a mixture of ^13^C-labeled internal standards (Cambridge Isotope Laboratories Inc., Andover, MA, USA) comprising PCB-28, PCB-52, PCB-101, PCB-118, PCB-138, PCB-153, PCB-156, and PCB-180, HCB, and *p,p*′-DDE and kept overnight at 4°C. Before extraction, we thawed the spiked samples at room temperature, added a 4:1 (vol/vol) mixture of formic acid and *iso*-propanol (J.T. Baker, Phillipsburg, NJ, USA), and sonicated them. Extraction was performed with five 6-mL aliquots of *n*-hexane (Merck KGaA, Darmstadt, Germany) by manual shaking, followed by centrifugation at 3,500 revolutions per minute (rpm) for 5 min. We removed the *n*-hexane aliquots collected from centrifugation, pooled them in a centrifuge tube, and carefully concentrated them. Concentrated sulfuric acid (H_2_SO_4_, Carlo Erba, Milan, Italy) was added to the *n*-hexane extracts; the two phases were vigorously shaken and then separated by centrifugation (3,500 rpm for 20 min). We reduced the volume of the purified extracts and transferred them into 1-mL vials to undergo instrumental analysis.

Instrumental analysis was carried out by using ion trap mass spectrometry (IT-MS) (Polaris Q; Thermofisher Scientific Inc., Waltham, MA, USA) coupled with high-resolution gas chromatography (GC). A RTX-5MS 60-m length, 0.25-mm i.d. capillary column (Restek Corporation, State College, PA, USA) coated with a 0.25-μm film was employed to separate the extracted compounds. The initial oven temperature of 70°C was increased to 190°C at a rate of 30°C/min, subsequently to 280°C at 5°C/min and to 330°C at 20°C/min, and maintained at 330°C for 4 min (total GC run time was 28 min). The injector was operated in the splitless mode with a 1.5-min splitless time. The initial injector temperature of 70°C was increased to 280°C at a rate of 14.5°C/sec. The transfer line and the ion source temperatures were set at 290 and 250°C, respectively. The IT-MS detector was operated in the electron ionization mode (70 eV) and MS/MS mode. Selected daughter ions used for quantification were (analyte, daughter ion): PCB-28, PCB-186; PCB-52, PCB-257; PCB-101, PCB-291; PCB-105, PCB-256; PCB-118, PCB-256; PCB-138 and PCB-153, PCB-325; PCB-156 and PCB-167, PCB-290; PCB-170 and PCB-180, PCB-361; HCB-249; *p,p*′-DDE-248. Data were processed using the XCALIBUR software (Thermofisher Scientific Inc.). Based on the ^13^C-labeled compounds employed, recovery ranges were within 75–110% for all compounds. Analytic reliability was warranted by the use of an in-house validated method (Ingelido et al. 2008). The laboratory has a consolidated experience in the analysis of halogenated organic micro-contaminants and periodically participates in interlaboratory exercises concerning the analysis of PCDDs, PCDFs, PCBs, organo-chlorinated pesticides, and some brominated flame retardants in dietary, biological, and environ mental matrices.

### Analysis of serum samples: compounds with dioxin-like activity

We conducted the analysis of compounds eliciting dioxin-like activity by the dioxin receptor (DR or AhR)-driven chemically activated luciferase expression bioassay (DR-CALUX; BioDetection Systems, Amsterdam, The Netherlands), a bioanalytic tool used to detect AhR active compounds, such as dioxins, present in different environmental and biological matrices (Hoogenboom et al. 1999; Murk et al. 1997).

DR-CALUX cells were purchased from BioDetection Systems. We cultured them routinely in a 5% CO_2_ atmosphere at 37°C in Alpha MEM supplemented with 10% heat-inactivated fetal calf serum (GIBCO BRL, Gaithersburg, MD, USA). The luciferase assay system, Britelite kit, was purchased from PerkinElmer Life and Analytical Science (Boston, MA, USA).

Analysis involved liquid-liquid extraction of blood serum with a 97:3 (vol/vol) mixture of *n*-hexane and diethyl ether and lipid removal by eluting the extract on a silica gel column concentrated H_2_SO_4_. The purified extract was quantitatively transferred to a vial, evaporated, and dissolved in dimethyl sulfoxide (DMSO) for DR-CALUX measurement. DR-CALUX cells were seeded in 96-multi-well plates (Packard ViewPlate, PerkinElmer) and incubated for 24 hr in a CO_2_ incubator. We performed the cell treatments in triplicate, adding to each well 100 μL of sample extract diluted in culture medium just before use, using DMSO as vehicle (0.5%, vol/vol DMSO in culture medium). After 24 hr of incubation at 37°C, cell monolayers were checked under an inverted microscope to exclude any cytotoxic effects of the extracts. We removed the exposure medium, washed the cells with phosphate-buffered saline with calcium and magnesium (pH 7.4), and lysed them with 100 μL Britelite solution. Light production was immediately measured with a MicroBeta luminescence counter (MicroBeta Jet, 1450 LSC, PerkinElmer). We always included a standard calibration curve of 2,3,7,8-TCDD in the experiments and simultaneously analyzed it with the samples (Sanderson et al. 1996; Windal et al. 2005). Eight 2,3,7,8-TCDD concentrations (0.3–300 pM/well), dissolved in DMSO and processed as described for sample extracts, were tested. Results expressed as relative light unit values were transformed into 2,3,7,8-TCDD toxicity equivalents (TEQs) using the BioDetection Excel file. Results were expressed in picograms of DR-CALUX-TEQs (C-TEQs) per gram of serum fat (pgC-TEQs/g fat). The limit of detection was calculated as the signal measured from the DMSO solvent control on each well plate plus three times its standard deviation.

### Lipid analysis

Concentrations of total cholesterol, phospholipids, and triglycerides were determined by enzymatic methods (Ingelido et al. 2008) and the use of colorimetric kits (Futura System s.r.l., Rome, Italy).

### Sample size

In setting the study design, sample size estimation was performed to determine the number of women per group sufficient to detect a true odds ratio (OR) between 2.5 and 3.0. With a power of 80%, type 1 error of 5%, and 0.20 probability of exposure in controls, we calculated that 64–94 subjects (ORs = 3.0 and 2.5, respectively) were required per group. A retrospective power analysis based on 80 cases and 78 controls enrolled in the study and a significance level of α set to 5% provided a power estimation of 72.6% to detect a risk of 2.5 and 88% to detect a risk of 3.0.

### Statistical analysis

Serum concentrations of all the analytes determined were subjected to statistical analysis. Differences in analyte levels between groups were investigated; geometric rather than arithmetic means were employed. The statistical significance of differences between cases and controls was assessed by Student’s *t*-test.

The concentration distribution of each analyte was divided by tertiles, and women were classified at low, medium, and high exposure. Using unconditional logistic regression analysis to adjust for potential confounders, the adjusted ORs and 95% confidence intervals (CIs) were estimated for the second and third tertile of PCBs, C-TEQs, HCB, and *p,p*′-DDE. Variables included in the final model were age (years), BMI (kilograms per square meter), smoking habits (nonsmokers, exsmokers, smokers), and evidence of relevant weight modifications in the last 5 years (> 10 kg). In addition, with the aim to investigate whether different histopathologic features of endometriosis were related to different etiologic risk factor patterns, patients were classified according to the presence of ovarian endometrioma, peritoneal lesion, and deep lesions, and the groups were compared. The relationship between the severity of disease, according to the revised American Society of Reproductive Medicine (ASRM) classification and the detected chemical levels was assessed by analysis of variance and chi-square test. Multiple linear regression analysis was used to test the association between log-transformed PCB concentrations in serum and dietary habits (milk, meat, fish) after checking for potential confounders.

## Results

A total of 158 women were enrolled in the study, 80 cases and 78 controls. Among cases, there were eight patients with stage I endometriosis, 5 with stage II, 44 with stage III, and 23 with stage IV. In particular, deep endometriotic nodules were present in 6 women (7.5%), ovarian endometriomas were observed in 72 patients (90.0%), and peritoneal implants in 45 cases (56.2%) (Table 1). The control group consisted of 78 women with other benign gynecologic conditions, including eight uterine myomas and 70 adnexal masses, with no laparoscopic evidence of endometriosis and random peritoneal biopsies negative for the disease at histologic analysis.

The main characteristics of the studied population, as derived from the administered questionnaires, are shown in Table 2. Mean age at interview was slightly higher in cases (31.6 ± 6.0 years) compared with controls (29.5 ± 6.1 years). Moreover, cases had a lower BMI (21.1 ± 2.8) than controls (22.4 ± 4.6). Both these differences were taken into account using a multivariate analysis. Age at menarche, having been breastfed, smoking habits, and alcohol consumption did not significantly differ between the groups. Dietary habits, described by the analysis of the dietary intakes of the main categories of food-stuffs, were not significantly different in the case of meat and fish. Average monthly consumption of milk and dairy products was slightly different among cases and controls (41 vs. 33 times/ month), but the associated incremental risk of endometriosis could be considered negligible (OR = 1.02; 95% CI, 1.00–1.04).

Geometric means and pertinent CIs of the analytes assessed in cases and controls are shown in Table 3. The concentrations of all the analyzed PCBs were higher in cases than in controls. Significant differences (*p* < 0.05) were found for PCB-101, PCB-156, and PCB-170, and highly significant differences (*p* < 0.01) were found for PCB-52, PCB-118, PCB-138, PCB-153, and PCB-180. *p,p*′-DDE and total PCB serum concentrations, as the sum of all the 11 congeners analyzed, were also significantly higher (relative increase: 44.9 and 48.4%, respectively) in cases than in controls.

As for the AhR ligands that elicit a response of the DR-CALUX system [dioxins, DL-PCBs, and possibly other (pseudo) planar aromatic pollutants present in serum], no significant difference was detected between cases and controls, despite the observed increase of two DL-PCBs (PCB-118, *p* = 0.0002, and PCB-156, *p* = 0.01) in women with endometriosis. However, as noted above, C-TEQs account for all compounds with dioxin-like activity, with PCBs 118 and 156 likely providing a minor contribution.

Table 4 shows the frequency distributions of all the analytes divided by tertiles, as well as the results of the multivariate analysis adjusted for age at interview, smoking habits, BMI, and weight loss. Concentrations of PCB congeners 118, 138, 153, and 170 are associated with a significant increased risk of endometriosis for the second and third tertile when compared with the lowest tertile. Risk of endometriosis appears to be significant (OR = 3.05; 95% CI, 1.25–7.42) also for the highest serum concentrations of PCB-180 (≥ 60.5 ng/g fat).

As to the sum of all PCB congeners, patients in the mid and upper tertiles have a 4- to 5-fold increased risk of having the disease.

Covariate-adjusted ORs showed that no increased risk of endometriosis was associated with serum levels of HCB, *p,p*′-DDE, and total C-TEQs, the latter determined by the DR-CALUX bioassay.

To explore a possible correlation between analyte serum concentrations and the disease type and localization, we stratified cases by peritoneal, deep, or ovarian endometriosis and compared levels of total PCBs, C-TEQs, HCB, and *p,p*′-DDE among groups. No differences in mean analyte levels could be found in women with endometriosis with respect to the different kind of disease.

Furthermore, the association between serum concentrations of the analyzed organo-chlorinated compounds and the stage of the disease (according to the revised ASRM classification) was investigated. No significant differences between groups were observed: Data analysis showed that pollutant concentrations did not correlate with disease severity.

The relationship between different food intakes and values of PCBs, C-TEQs, HCB, and *p,p*′-DDE was estimated by a multivariate regression model. The lack of significance (*p* > 0.05) of slope coefficients suggests that food intake is not associated with the concentrations of the chemicals analyzed in the present study.

## Discussion

The results of the present study are in agreement with findings we had obtained in two previous investigations carried out on smaller samples of nulliparous women, in which cases resulted to have a significant increase in blood concentrations of PCB-118, PCB-138, PCB-153, PCB-180, the sum of the mentioned 11 PCBs (Porpora et al. 2006; Quaranta et al. 2006), as well as of *p,p*′-DDE (Quaranta et al. 2006). In particular, a greater than 3-fold increase was found for these four PCB congeners and their sum.

The observed absence of an increase in TEQ values in women with endometriosis with respect to controls confirms the results we obtained in a study carried out on pooled blood samples from two small groups of Italian and Belgian women of reproductive age (De Felip et al. 2004), in which no correlation was observed between dioxin-like compounds and the disease, on a country basis. This agreement is observed although different analytical methodologies were used: High-resolution GC coupled with high resolution mass spectrometry was employed to determine dioxin-like compounds in our previous study, whereas DR-CALUX bioassay was used in this work.

The present study and the three above have similar study designs. In fact, with the exception (in this study) of a small minority of non-nulliparous women who had never breastfed, only nulliparous women were enrolled, to avoid the confounding factor of breast-feeding, known to determine a significant decrease in organochlorine body burden. All women underwent surgical confirmation of the disease or its absence, and only women with no visual evidence or histologic signs of endometriosis in random peritoneal biopsies were included in the control group. In the present study, women with complaints of infertility were not enrolled as controls, because some organochlorinated pollutants have been hypothesized to be associated with infertility, in particular *p,p*′-DDE (Korrick et al 2001; Weiss et al. 2006). There are no data regarding a correlation between the other gynecologic conditions of the control group (uterine myomas, benign adnexal mass) and the aforesaid pollutants.

A comparison of this study with other case–control studies designed to explore the association between persistent organo-halogenated pollutants and endometriosis is complicated by the variety of study designs, analytical methodologies used, and number and type of compounds or congeners assessed. Recent papers (Anger and Foster 2008; Heilier et al. 2008) have presented a comprehensive overview of the studies carried out on this topic, the vast majority focused on assessing the association between dioxins and/or PCBs and peritoneal endometriosis, and have discussed their limited or null comparability. On the whole, no significant correlation was observed in case–control studies between NDL-PCBs and/or DL-PCBs and endometriosis (Buck Louis et al. 2005; Fierens et al. 2003; Gerhard and Runnebaum 1992; Heilier et al. 2004, 2005; Lebel et al. 1998; Pauwels et al. 2001; Tsukino et al. 2005), although a nonsignificant increase of the most abundant NDL-PCB-138, PCB-153, and PCB-180 was observed in one study (Gerhard and Runnebaum 1992) and of the same three congeners plus the DL-PCB-118 in another study (Pauwels et al. 2001).

As to the sparse case–control studies available specifically focused on the AhR ligands (PCDDs, PCDFs, and DL-PCBs), a significant association with these chemicals and the disease was generally not observed (Anger and Foster 2008; Pauwels et al. 2001), whereas a few studies reported increased but not significant ORs for the disease (Buck Louis et al. 2005; Heilier et al. 2005; Mayani et al. 1997). In one study that considered peritoneal endometriosis and deep endometriotic (adenomyotic) nodules separately (Heilier et al. 2005), a significantly increased risk was associated with dioxin and DL-PCB serum concentrations in women with deep endometriosis.

As to the association between endometriosis and organohalogenated pollutants other than PCBs and/or dioxins, only a few studies are available, carried out on PBBs (Hoffman et al. 2007) or organochlorinated pesticides, including HCB and *p,p*′-DDE (Tsukino et al. 2005). No evidence of an association was found between endometriosis and PBB or organochlorinated pesticide serum levels in these studies.

In our study, no correlation was observed between the disease and HCB, a ubiquitous persistent pollutant identified in human tissues worldwide, although its use was discontinued decades ago. The effects of HCB on ovarian function and circulating ovarian steroids were demonstrated on exposed nonhuman primates (Foster et al. 1995).

With regard to *p,p*′-DDE, the main metabolite of the pesticide *p,p*′-DDT characterized by both immune and endocrine toxicity (Halloway 2005; Wójtowicz et al. 2007), findings from the present investigation confirm an increase in serum concentrations in women with endometriosis observed in a previous investigation. In that study, on the basis of the reported observation of an immunologic dysregulation in women simultaneously exposed to *p,p*′-DDE and PCBs (Daniel et al. 2002), we evaluated the immunologic status of two small groups of women and determined the serum concentrations of the most abundant PCBs and *p,p*′-DDE to evaluate their possible role in dysregulation of the immune function observed in patients with endometriosis. The results we obtained showed that peripheral blood NK cytotoxic activity and interleukin-1 beta and interleu-kin-12 production were significantly down-regulated in patients with endometriosis with respect to controls, and this matched with higher serum concentrations of PCBs and *p,p*′-DDE in the same patients (Quaranta et al. 2006).

In addition to peritoneal endometriosis, deep endometriosis (adenomyotic) nodules of the rectovaginal septum, considered a distinct clinical entity by some authors (Donnez et al. 1996), have also been studied as to their potential association with exposure to organo-chlorinated pollutants. For this type of endometriosis, Heilier and coworkers (Heilier et al. 2004, 2005) found a significantly increased risk associated with both NDL-PCBs and dioxin-like compounds in serum. When we analyzed the association between the serum concentrations of PCBs and pesticides and the different types of disease, no differences were found between cases with peritoneal implants, ovarian endometrioma, deep lesions, or combined lesions. Therefore, it can be hypothesized that PCBs and *p,p*′-DDE are risk factors for developing any kind of endometriosis. Regarding the relationship between the severity of disease (according to the ASRM classification) and exposure to organochlorinated compounds, we did not find any significant correlation between the pollutant serum concentrations and the different stages of the disease.

The reason for the observed increase in PCB and *p,p*′-DDE serum concentrations in patients with endometriosis remains to be elucidated. Because no correlation with dietary habits was found, such an increase could be associated with a different capacity in bio-activation and/or detoxication due to both genetic makeup and/or induction/inhibition phenomena in the tested population. Work is in progress on genetic polymorphisms of glutathione *S*-transferase and cytochrome P-450, enzymes involved in organochlorine compound biotransformation, to explore the gene–environment interactions as a possible cause of the observed higher levels of the aforesaid compounds in patients with endometriosis and as a risk factor for the disease onset/progression, as suggested by some studies (Ertunc et al. 2005; Hsieh et al. 2004; Tsuchiya et al. 2007).

In summary, our data show that exposure to PCBs and *p,p*′-DDE represents a risk factor for endometriosis. In particular, the observation that ORs increase with increasing PCB concentrations strongly supports the hypothesis of an association between exposure to these chemicals and the disease, although the specific mechanisms of actions remain to be characterized.

## Figures and Tables

**Table 1:** Laparoscopic findings and stage of endometriosis classified according to rASRM (1997) in women affected by endometriosis (cases).

Laparoscopic findings	Cases [no. (%)]
Stage of endometriosis (rASRM)	
I	8 (10.0)
II	5 (6.2)
III	44 (55.0)
IV	23 (28.7)
Ovarian endometrioma	
Yes	72 (90.0)
No	8 (10.0)
Peritoneal lesions	
Yes	45 (56.2)
No	35 (43.7)
Type of peritoneal lesion	
Typical	23 (53.5)
Subtle	6 (13.9)
Both	14 (32.6)
Deep endometriosis	
Yes	6 (7.5)
No	74 (92.5)

**Table 2:** Sociodemographic characteristics of cases (*n* = 80) and controls (*n* = 78).

Characteristics	Controls	Cases	*p-*Value^a^
Age [years (mean ± SD)]	29.5 ± 6.1	31.6 ± 6.0	0.03
Age (years)			
≤ 25	18 (23.1)	12 (15.0)	
26–35	46 (59.0)	46 (57.5)	
≥ 36	14 (17.9)	22 (27.5)	0.23
No. of children^b^			
0	74 (94.9)	74 (96.1)	
1	4 (5.1)	2 (2.6)	
2	0 (—)	1 (1.3)	0.99
BMI (mean ± SD)	22.4 ± 4.6	21.1 ± 2.8	0.03
Relevant weight modifications in the last 5 years (> 10 kg)^b^			
Yes	9 (11.5)	8 (10.1)	
No	69 (88.5)	71 (89.9)	0.98
Food consumption [times/month (mean ± SD)]			
Milk and dairy products	33 ± 16	41 ± 25	0.02
Meat	25 ± 10	25 ± 12	0.93
Fish	7 ± 5	8 ± 6	0.49
Age at menarche [years (mean ± SD)]	12.1 ± 1.4	12.2 ± 1.5	0.84
Breast-fed as children^b^			
Yes	55 (70.5)	56 (70.9)	
No	13 (16.7)	15 (19.0)	
Unknown	10 (12.8)	8 (10.1)	0.83
Smoking status			
Nonsmokers	41 (53.9)	44 (55.0)	
Ex-smokers	7 (9.2)	13 (16.2)	
Smokers (cigarettes/day)			
1–9	8 (10.5)	7 (8.8)	
10–19	13 (17.1)	8 (10.0)	
≥ 20	7 (9.3)	8 (10.0)	0.54

Values shown are mean ± SD or no. (%)

a Student’s t- test, chi-square test, Fisher test.

b Numbers do not add up due to missing values.

**Table 3:** Serum concentrations [geometric mean (95% CI); ng/g fat] of PCBs, C-TEQs, HCB, and *p,p*′-DDE in cases and controls.

	Controls	Cases	*p-*Value^a^
PCB-28	3.4 (2.5–4.5)	4.3 (3.1–6.0)	0.3
PCB-52	1.6 (1.3–1.9)	2.2 (1.9–2.6)	< 0.01
PCB-101	1.6 (1.3–1.9)	2.1 (1.7–2.5)	0.04
PCB-105	5.7 (4.1–8.1)	6.8 (4.9–9.4)	0.47
PCB-118	15.2 (13.1–17.6)	23.4 (19.7–27.9)	< 0.01
PCB-138	33.8 (29.0–39.3)	50.7 (43.4–59.1)	< 0.01
PCB-153	61.8 (51.8–73.8)	99.8 (87.5–113.8)	< 0.01
PCB-156	5.7 (4.7–6.9)	8.3 (6.7–10.4)	0.01
PCB-167	2.5 (2.0–3.1)	3.2 (2.6–3.9)	0.08
PCB-170	6.1 (4.8–7.7)	8.8 (7.1–10.8)	0.02
PCB-180	34.4 (29.0–40.8)	48.6 (42.0–56.2)	< 0.01
Total PCBs	203.0 (179.4–229.8)	301.3 (271.1–334.9)	< 0.01
(pgC-TEQs/g fat)	20.9 (17.3–25.2)	18.6 (14.5–23.9)	0.47
HCB	40.9 (32.6–51.4)	35.8 (28.6–44.8)	0.41
*p,p*′-DDE	303.1 (247.0–371.9)	439.1 (360.4–534.9)	0.01

a *t*-Test on log-transformed values.

**Table 4:** Frequency distribution of serum levels of PCB, C-TEQs, HCB, and *p,p*′-DDE and adjusted ORs for endometriosis by tertiles.

Concentration (ng/g fat)	Controls (%)	Cases (%)	OR_adj_^a^ (95% CI)
PCB-28			

≤ 2.3	29.49	37.50	1.0
2.4–6.5	43.59	25.00	0.35 (0.15–0.84)
≥ 6.9	26.92	37.50	0.98 (0.42–2.25)

PCB-52			

≤ 1.4	41.03	27.50	1.0
1.5–2.4	35.90	35.00	1.51 (0.68–3.35)
≥ 2.5	23.08	37.50	1.96 (0.84–4.56)

PCB-101			

≤ 1.35	39.74	30.00	1.0
1.36–2.4	34.62	30.00	1.29 (0.57–2.90)
≥ 2.5	25.64	40.00	1.93 (0.86–4.36)

PCB-105			

≤ 3.7	35.90	31.25	1.0
3.8–11.2	34.62	32.50	1.23 (0.55–2.75)
≥ 11.3	29.49	36.25	1.55 (0.68–3.50)

PCB-118			

≤ 13.2	48.72	21.25	1.0
13.3–24.2	25.64	38.75	3.17 (1.36–7.37)
≥ 24.3	25.64	40.00	3.79 (1.61–8.91)

PCB-138			

≤ 33.6	46.15	21.25	1.0
33.7–56	29.49	36.25	2.37 (1.02–5.48)
≥ 57	24.36	42.50	3.78 (1.60–8.94)

PCB-153			

≤ 62	51.28	16.25	1.0
63–104	24.36	42.50	4.64 (1.93–11.1)
≥ 105	24.36	41.25	4.88 (2.01–11.0)

PCB-156			

≤ 4	39.74	27.50	1.0
5–9	34.62	33.75	0.98 (0.43–2.24)
≥ 10	25.64	38.75	1.65 (0.71–3.82)

PCB-167			

≤ 1.9	35.90	31.25	1.0
2–3.9	34.62	32.50	0.97 (0.43–2.17)
≥ 4	29.49	36.25	1.18 (0.50–2.77)

PCB-170			

≤ 5.37	45.95	21.25	1.0
5.38–12.4	29.73	37.50	2.71 (1.13–6.51)
≥ 12.5	24.32	41.25	3.52 (1.41–8.79)

PCB-180			

≤ 33.2	43.24	23.75	1.0
33.3–60.4	35.14	32.50	1.33 (0.57–3.11)
≥ 60.5	21.62	43.75	3.05 (1.25–7.42)

Total PCBs			

≤ 208	51.35	16.25	1.0
209–305	25.68	41.25	4.64 (1.93–11.16)
≥ 306	22.90	42.50	5.63 (2.25–14.10)

(pgC-TEQs/g fat)			

≤ 15.6	30.2	35.7	1.0
15.7–29.5	35.8	30.4	0.52 (0.18–1.48)
≥ 29.6	34.0	33.9	0.73 (0.26–2.01)

HCB			

≤ 31	35.90	33.75	1.0
32–54	30.77	35.00	0.91 (0.40–2.08)
≥ 55	33.33	31.25	0.65 (0.27–1.54)

*p,p*′-DDE			

≤ 231	41.03	26.25	1.0
232–492	30.77	35.00	1.54 (0.66–3.58)
≥ 493	28.21	38.75	2.14 (0.93–4.93)

a OR adjusted for age, smoking habits, BMI, evidence of weight modification.
